# Facteurs associés à la dissociation immunovirologique chez les patients infectés par le VIH-1 sous traitement antirétroviral hautement actif au Centre de Traitement Ambulatoire (CTA) de Dakar

**DOI:** 10.11604/pamj.2017.27.16.9811

**Published:** 2017-05-08

**Authors:** Daye Kà, Noël Magloire Manga, Ndéye Fatou Ngom-Guéye, Diop Ndiaga, Moustapha Diop, Viviane Marie Pierre Cisse-Diallo, Khardiata Diallo-Mbaye, Ndèye Aissatou Lakhe, Louise fortès-Déguenonvo, Cheikh Tidiane Ndour, Sylvie Audrey Diop-Nyafouna, Moussa Seydi

**Affiliations:** 1 Service des Maladies Infectieuses et Tropicales de Fann, Université Cheikh Anta Diop de Dakar, Sénégal; 2 UFR Santé – Université de Ziguinchor Sénégal; 3 UFR Santé, Université de Thiès Sénégal

**Keywords:** Dissociation, immunovirologique, VIH, Dakar, Dissociation, immunovirologic, HIV, Dakar

## Abstract

**Introduction:**

L'objectif de ce travail était d'évaluer les différents facteurs associés à la dissociation immunovirologique malgré un traitement antirétroviral hautement actif et efficace.

**Méthodes:**

Il s'agissait d'une étude de cohorte historique, descriptive et analytique faite à partir de dossiers de patients infectés par le VIH-1; sous traitement antirétroviral depuis au moins 12 mois, suivis dans la cohorte du CTA de 2001 à 2011 et ayant une charge virale indétectable depuis 6 mois.

**Résultats:**

Durant cette période d'étude de 10 ans, la prévalence de la DIV était de 19,3%. Le sexe féminin était prédominant avec un sexe ratio de 1,9. La dissociation immunovirologique a été plus fréquemment rencontrée chez les patients de sexe masculin (29,7% vs 14,1%) avec une différence statistiquement significative (p = 0,00006). L'âge médian était de 44 ans ± 10 ans. Un antécédent de tuberculose a été retrouvé dans environ un tiers des cas (31,4%). La dissociation immunovirologique était significativement plus fréquente chez les patients ayant un antécédent de tuberculose (p = 0,00005). La plupart des patients (68%) était au stade SIDA 3 ou 4 de l'OMS. Les patients ayant une dissociation immunovirologique étaient plus souvent aux stades 3 et 4 de l'OMS (p = 0,0001). La dénutrition a été notée dans plus de la moitié des cas (56,2%) et la dissociation immunovirologique prédominait chez les patients dénutris (p=0,005). Le taux moyen de lymphocytes TCD4+ était de 86,7± 83 cellules / mm^3^. La dissociation immunovirologique était plus fréquente chez les patients ayant un taux de lymphocytes TCD4 bas à l'initiation avec une différence statistiquement significative (p = 0,00000). En analyse multivariée; Seuls l'âge supérieur ou égal à 43 ans, le taux de CD4 initial < 100 c/mm^3^ et le sexe masculin étaient significativement associés à cette dissociation immunovirologique.

**Conclusion:**

Les principaux facteurs associés à la dissociation immunovirologique étant évalués, d'autres études portant sur ce groupe mériteraient d'être envisagées afin de connaitre l'impact de cette réponse immunologique partielle sur la survenue d'infections opportunistes ou bien la mise en place d'une trithérapie spécifique uniquement dans le but d'avoir une restauration immunologique optimale.

## Abstract (en)

**Introduction:**

The objective of this work is to evaluate the different factors associated with immunovirologic dissociation despite highly active and effective antiretroviral treatment.

**Methods:**

We conducted a retrospective, cohort, descriptive and analytical study of the medical records of HIV-1 infected patients having received at least 12 months of antiretroviral therapy, followed in the ATC cohort from 2001 to 2011 and with undetectable viral load in the last 6 months.

**Results:**

During this 10-year study period, the prevalence of IVD was 19.3%. Female sex was predominant, with a sex ratio of 1.9. Immunovirologic dissociation was more frequent in male patients (29.7% vs 14.1%) with a statistically significant difference (p = 0,00006). The average age was 44 years ± 10 years. A history of tuberculosis was found in about a third of the cases (31.4%). Immunovirologic dissociation was significantly more frequent in patients with a history of tuberculosis (p = 0.00005). Most patients (68%) had AIDS at WHO clinical stages 3 or 4. Patients with immunovirologic dissociation were more often in WHO clinical stages 3 and 4 (p = 0.0001). More than half of the cases (56.2%) were found to be malnourished and immunovirologic dissociation was prevalent in malnourished patients (p=0.005). The mean CD4+ T lymphocytes counts was 86.7± 83 cells / mm^3^. Immunovirologic dissociation was more frequent in patients with initial low CD4+ T lymphocyte counts and with a statistically significant difference (p = 0.00000). By multivariate analysis, only age greater than or equal to 43 years, CD4 initial counts < 100 c/mm^3^ and male sex were significantly associated with this immunovirologic dissociation.

**Conclusion:**

Our study assessed the main factors associated with immunovirologic dissociation. Other studies of this nature would also merit consideration in order to highlight the impact of this partial immune response on the emergence of opportunistic infections or the implementation of a specific tritherapy for the sole purpose of producing fully successful immune restoration.

## Introduction

L'infection par le VIH, d'une maladie constamment létale, est devenue depuis la fin des années 90, une maladie chronique persistante grâce aux molécules antirétrovirales qui permettent de contrôler la réplication virale. Elle reste néanmoins un problème majeur de santé publique avec 34 millions de personnes infectées dans le monde et représente l'une des premières causes de décès à cette échelle [1, 2]. Les antirétroviraux (ARV) inhibent efficacement la réplication virale et favorisent la restauration immunitaire qui met les personnes infectées à l'abri des infections opportunistes majeures. L'objectif principal du traitement antirétroviral est de rendre la charge virale plasmatique indétectable (< 50 copies/ml), ce qui permet une meilleure restauration immunitaire et limite au maximum le risque de sélection de virus résistants. Des études ont démontré que les patients infectés par le VIH avec une restauration des lymphocytes CD4 à un taux ≥ 500/mm^3^ sous trithérapie avaient une espérance de vie comparable à celle de la population générale. Cependant certains patients infectés par le VIH/sida sous traitement antirétroviral présentent une réponse immunitaire partielle avec un taux de Lymphocytes T CD4+ < 200/mm^3^ malgré une charge virale plasmatique indétectable. Ce phénomène appelé dissociation immunovirologique (DIV) est de moins bon pronostic et peu étudié en Afrique. C'est dans ce contexte que nous avons effectué cette étude dont les objectifs sont de: déterminer la prévalence de cette dissociation immunovirologique; Identifier les différents facteurs associés à cette dissociation immunovirologique malgré un traitement antirétroviral hautement actif et efficace.

## Méthodes

**Type d'étude:** Il s'agissait d'une étude descriptive et analytique de cohorte historique, allant du 1^er^ janvier 2001 au 31 décembre 2011.

**Population d'étude:** Etaient inclus dans notre étude les patients: infectés par le VIH-1; sous traitement antirétroviral depuis au moins 12 mois; suivis dans la cohorte du CTA de 2001 à 2011: ayant une charge virale indétectable depuis 6 mois et dont les données sont dans la base de données Esope.

Nous n'avons pas inclus les patients dont les dossiers étaient incomplets et dont la charge virale et/ou le taux de LTCD4+ étaient indisponibles. La dissociation immunovirologique est définie comme étant un taux de Lymphocytes T CD4 + de moins de 200/mm3 après au moins 6 mois de traitement antirétroviral malgré un succès virologique (Charge virale indétectable) [3, 4].

**Variables étudiés:** Les variables étudiées étaient: les caractéristiques sociodémographiques notamment l'âge, le sexe, les antécédents; les caractéristiques biocliniques à savoir : le stade clinique OMS, la BMI, les données hématologiques, le taux de CD4, la charge virale.

**Analyse des données:** L'analyse des données a été faite avec le logiciel Epi Info version 3.5.3. Les comparaisons de proportion ont été faites avec le test du CHI-2. La régression logistique a été faite pour l'analyse multivariée des données. Les facteurs associés avec une valeur p < 0,05 ont été retenus.

**Aspect éthique:** Une base de données anonyme a été constituée à partir des dossiers médicaux et sociaux des patients du CTA. Aucune information ne permettait d'identifier les patients inclus dans cette étude. La base de données reste une propriété du CTA. L'étude a été autorisée par le chef de service des maladies infectieuses.

## Résultats

### Etude descriptive

**Aspects épidémiologiques:** Durant la période d'étude allant de Janvier 2001 à Décembre 2011, nous avons colligé 89 cas de dissociation immunovirologique sur un total de 460 dossiers consultés, soit une prévalence de 19,3%. L'âge moyen était de 44 ans ± 10 ans avec des extrêmes de 21 ans et 78 ans et le sexe féminin était prédominant avec un sexe ratio de 1,9. Un tiers des patients (31,4%) présentait un antécédent de tuberculose.

**Aspects cliniques:** La plupart des patients était au stade SIDA: OMS 3 et 4 (68%). La dénutrition (IMC inférieur à 18,5 kg/m^2^) a été notée dans plus de la moitié des cas des cas (56,18%) et l'IMC moyen était de 21 kg/m^2^ ± 4,3 (Tableau 1).

**Tableau 1:** Répartition des cas de dissociation immunovirologique selon le stade clinique OMS

Classification OMS	Effectif	Pourcentage (%)
Stade 1	10	11,2
Stade 2	18	20,2
Stade 3	41	46
Stade 4	20	22,5

**Aspects paracliniques:** La répartition des cas de DIV selon les résultats de la biologie est présentée dans le Tableau 2.

**Tableau 2:** Répartition des cas de DIV selon les résultats de la biologie initiale

Variables	Moyenne	Ecart-type	Médiane	Extrêmes
Taux CD4 (/mm ^3^)	± 86,7	83,4	65	2 - 407
Hémoglobine (g/dl)	± 10,5	2,0	10,7	3,7 - 15
Leucocytes (/mm^3^)	± 4630	1758	4400	700 -12500
Lymphocytes (/mm^3^)	± 1724	1171	1500	8 -14000
Neutrophiles (/mm^3^)	± 2657	1438	2301	44 -10625

### Etude analytique: facteurs associés à la DIV

**Analyse bivariée:** aAu plan épidémiologique, la dissociation immunovirologique était significativement différente selon le sexe (46 hommes soit 29,68% vs 43 femmes soit 14,10% avec un p égal à 0,00006. Elle était aussi plus fréquente chez les patients ayant un antécédent de tuberculose avec une différence statistiquement significative (p = 0,00005). Par contre, les patients présentant une dissociation immunovirologique avaient une moyenne d'âge plus élevée (46 ans vs 43 ans) mais sans différence statistiquement significative (p = 0,05). Au plan clinique, La dissociation immunovirologique prédominait chez les patients dénutris avec une différence statistiquement significative (p = 0,005). Cette dissociation immunovirologique était plus présente chez les patients au stade 3 et 4 de l'OMS (20 au stade 4 et 41 au stade 3) que chez ceux au stade 1 et 2 de l'OMS (18 au stade 2 et 10 au stade 1) avec une différence statistiquement significative (p = 0,0001). Au plan biologique, la dissociation immunovirologique était significativement plus fréquente chez les patients ayant un taux de CD4 initial bas (p=0,0000). Par contre cette différence n'était pas significative selon le taux d'hémoglobine.

**Analyse multivariée:** Après avoir ajusté sur toutes les autres variables indépendamment associées à la dissociation immunovirologique, seuls l'âge supérieur ou égal à 43 ans, le taux de CD4 initial < 100 cellules /mm^3^ et le sexe masculin demeurent significativement associés à cette dissociation immunovirologique à 12 mois de traitement ARV malgré une charge virale plasmatique contrôlée (Tableau 3).

**Tableau 3:** Analyse multivariée: facteurs associés à la dissociation immunovirologique

Variables	OR	IC à 95%	Valeur P
Age ≥ 43	2,02	1,17 – 3,54	0,0123
ATCD TB (= oui)	1,83	0,96 – 3,48	0,0647
LTCD4 initial < 100	4,17	2,45 – 7,09	0,0000
BMI < 18,5kg/m^2^	1,39	0,82 – 2,37	0,2229
Stade OMS 3 et 4	1,59	0,88 – 2,87	0,1202
Sexe (M/f)	1,76	1,03 – 2,99	0,0357

## Discussion

### Aspects épidémiologiques

Durant la période d'étude allant du 1e Janvier 2001 au 31 Décembre 2011, nous avons colligé 89 cas de dissociation immunovirologique sur un total de 460 dossiers consultés soit une prévalence de 19,3%. Ce taux était superposable à ceux retrouvés par Marimoutou et Grabar en France avec respectivement 16% et 17% [5, 6]. Il était nettement supérieur à celui retrouvé par Tan R. au Royaume Uni (8,7%) et Moore DM au Canada (15%) [7, 8]. Cependant, Lima au Canada, Anude au Nigéria et Manga au Sénégal avaient noté des taux nettement supérieurs respectivement 39%, 33% et 32,5% [9–11]. Ces différences seraient en partie dues à l'hétérogénéité des caractéristiques des cohortes notamment sur le plan des schémas thérapeutiques et des durées de traitement.

Le sexe féminin était prédominant avec un sexe ratio de 1,9. Ce qui est la règle dans la plupart des études réalisées en Afrique chez les patients vivant avec le VIH, dénotant ainsi la féminisation de l'épidémie [10, 12, 13]. Cependant Giordano TP aux USA et l'ART-CC avaient retrouvé une nette prédominance masculine [14, 15]. La dissociation immunovirologique a été plus fréquemment rencontrée chez les patients de sexe masculin (29,7% vs 14,1%) avec une différence statistiquement significative. Ce résultat est classiquement retrouvé dans la littérature comme le confirment les travaux de Giordano TP, Kelley CF aux Etats Unis et Viard JP en Europe [12, 14, 16]. En effet le sexe masculin est souvent associé à une mauvaise réponse immunitaire. Ceci s'explique selon certains auteurs, par le fait que la production thymique est plus importante chez le sujet de sexe féminin et elle est étroitement liée à la production de lymphocytes TCD4+ [12, 16, 17]. L'âge moyen était de 44 ans ± 10 ans avec des extrêmes de 21 ans et 78 ans. Cette moyenne d'âge était comparable à celle retrouvée par Kelley aux Etats Unis [12]. Cependant elle était supérieure à celles retrouvées par Manga à Dakar, Zannou au Bénin, Mouhari au Togo, avec respectivement un âge moyen de 39 ans, 37 ans et 35 ans [11, 18, 19]. Elle était plus élevée chez les patients ayant présenté une dissociation immunovirologique (p=0,05). Comme le confirment les études de DM Moore et al au Canada, de Kelley et Viard aux Etats Unis et de Manga à Dakar [8, 11, 12, 16].

L'âge avancé est souvent associé à une mauvaise réponse immunitaire. Ce phénomène est dû à la diminution de la production thymique avec l'âge du fait de l'involution du thymus avec une sécrétion ralentie de LT CD4+ [17]. La dissociation immunovirologique était significativement plus fréquente chez les patients ayant un antécédent de tuberculose (p = 0,00005). La tuberculose est elle-même cause d'immunodépression avec chute des LTCD4+ chez certains patients en dehors de toute co-infection avec le VIH.

#### Aspects cliniques

Sur le plan clinique, la dénutrition a été notée dans plus de la moitié des cas (56,2%) et la dissociation immunovirologique était significativement plus fréquente dans ce groupe (p=0,005). Ceci peut s'expliquer par le fait que la dénutrition soit associée à une diminution des fonctions immunitaires, surtout en cas de dénutrition protéique, où l'on observe une diminution du volume et de la fonction de tous les organes lymphoïdes, à commencer par le thymus. Ce déficit concerne l'immunité cellulaire, et les populations lymphocytaires. En cas de dénutrition protéique, la taille des populations de lymphocytes en particulier des LTCD4+ se réduit [20, 21]. La plupart des patients était au stade SIDA : OMS 3 et 4 (68%) et la dissociation immunovirologique prédominait significativement chez ces patients (p= 0,0001). En effet, ces stades correspondent à la survenue d'infections opportunistes graves qui apparaissent quand le taux de CD4 est inférieur à 200/mm3 [2, 22]. Ils traduisent une destruction importante des fonctions immunitaires et du système de restauration de l'immunité cellulaire. Ceci peut expliquer la mauvaise restauration immunitaire [12, 17].

#### Aspects paracliniques

Dans notre série, le taux moyen de lymphocytes TCD4+ était de 86,7 ± 83cellules / mm^3^. Ce taux bas de CD4+, retrouvé dans notre étude témoigne du dépistage tardif de l'infection à VIH dans nos régions. Il est inférieur à celui retrouvé par d'autres auteurs [12, 23]. La dissociation immunovirologique était plus fréquente chez les patients ayant un taux de lymphocytes TCD4 plus bas à l'initiation avec une différence statistiquement significative (p= 0,00000). Ce résultat est superposable aux données de la littérature, illustrées par les travaux de Kelley et Pinzone [12, 17]. En effet l'immunodépression sévère (CD4+ < 200/mm^3^), avant l'initiation d'un traitement ARV est un facteur favorisant la mauvaise réponse immunitaire [8, 17, 23, 24].

#### Facteurs associés à la dissociation immunovirologique

En analyse multivariée, seuls l'âge supérieur ou égal à 43 ans, le taux de CD4 initial < 100 cellules /mm^3^ et le sexe masculin demeurent significativement associés à cette dissociation immunovirologique à 12 mois de traitement ARV malgré une charge virale plasmatique contrôlée. Dans l'étude d'Anude au Nigéria [10], les facteurs associés à un défaut de restauration immunitaire, étaient: le sexe masculin (OR 1,80 ; IC95% : 1,13-2,88) avec une différence significative (p=0,03); l'âge inférieur à 30 ans (OR 1,50 ; IC95% : 1,11- 2,39) avec une différence statistiquement significative (p=0,03).

## Conclusion

Dans notre étude nous avons identifié comme facteurs associés à la dissociation immunovirologique : l'âge supérieur ou égal à 43 ans, le taux de CD4 initial < 100 cellules /mm^3^ et le sexe masculin. Il serait alors nécessaire de réaliser d'autres études portant sur ce groupe afin de connaitre l'impact de cette réponse immunologique partielle sur la survenue d'infections opportunistes ou bien la mise en place d'une trithérapie spécifique uniquement dans le but d'avoir une restauration immunologique optimale.

### Etat des connaissances actuelle sur le sujet

- La dissociation immunovirologique est un phénomène relativement fréquent, surtout dans les pays en développement du fait du dépistage tardif de l'infection à VIH;
- Les principaux facteurs favorisants, classiquement retrouvés, sont: le taux de lymphocytes T CD4+ bas à l'initiation, l'âge avancé et le sexe masculin entre autres.

### Contribution de notre étude à la connaissance

- Notre travail, en plus des facteurs déjà connus, a permis d'identifier d'autres facteurs associés à cette dissociation immunovirologique, notamment l'âge inférieur inférieur à 30 ans, la dénutrition et la présence d'un antécédent de tuberculose.

## Conflits d’intérêts

Les auteurs ne déclarent aucun conflits d’'intérêts.
